# A Homozygous SLC39A8 p.Asn369Thr Variant in an Infant With Leigh‐Like Encephalopathy

**DOI:** 10.1155/crig/4037228

**Published:** 2026-11-15

**Authors:** Muhammad Nasheet Sagri, Abida Akbar, Bushra Rehman, Zohra Hasan, Salman Kirmani, Khairunnisa Muhktiar

**Affiliations:** ^1^ Department of Pediatrics & Child Health Aga Khan University Karachi Pakistan aku.edu

**Keywords:** basal ganglia, congenital disorder of glycosylation, infantile encephalopathy, Leigh-like syndrome, manganese transporter, SLC39A8

## Abstract

**Background:**

Biallelic variants of SLC39A8 cause congenital disorders of glycosylation Type IIn (CDG‐IIn, OMIM #616721), a rare autosomal recessive condition characterized by impaired manganese (Mn) transport, defective glycosylation, and secondary mitochondrial dysfunction. Affected infants present with developmental delays, hypotonia, seizures, and symmetric basal ganglia lesions on brain MRI.

**Case Presentation:**

An 11‐month‐old girl, previously developmentally appropriate, presented with new‐onset seizures and regression of motor and social milestones. Examination revealed global hypotonia, poor feeding, and faltered growth. Brain MRI demonstrated bilateral, symmetric T2/FLAIR hyperintensities with restricted diffusion in the caudate nuclei, putamin, globi pallidi, subthalamic nuclei, and cerebral peduncles, with sparing of the dentate nuclei and no contrast enhancement. Whole exome sequencing identified a novel homozygous SLC39A8 variant, NM_001,135,146.2:c.1106A > *C* (p.Asn369Thr). Each parent was a heterozygous carrier, confirming autosomal recessive inheritance. This variant is absent from ClinVar and gnomAD and is currently classified as a variant of uncertain significance (VUS). The clinical phenotype and neuroimaging pattern are compatible with SLC39A8‐CDG; however, the identified variant remains a VUS, and biochemical confirmation could not be obtained because the relevant tests were unavailable. Confirmation would require functional studies. Supportive testing would include transferrin glycosylation analysis and blood manganese measurement; a normal conventional transferrin result alone would not exclude the diagnosis. If SLC39A8‐CDG is confirmed, manganese and galactose supplementation should be considered only under specialist metabolic supervision with appropriate biochemical monitoring. This case suggests a possible phenotype‐genotype association but does not establish the pathogenicity of p.Asn369Thr.

## 1. Background

Congenital disorders of glycosylation (CDGs) are a heterogeneous group of inborn errors of metabolism characterized by defective glycoprotein and glycolipid biosynthesis [1]. SLC39A8‐CDG (CDG Type IIn) results from biallelic loss‐of‐function variants in SLC39A8, which encodes the divalent metal transporter ZIP8 that mediates cellular uptake of Mn and Zn [1, 2]. Loss of ZIP8 function causes severe intracellular manganese deficiency, impairing Mn‐dependent Golgi glycosyltransferases (notably *β*‐1,4‐galactosyltransferase and MGAT3) and mitochondrial manganese superoxide dismutase (MnSOD) [3–5]. This dual disruption of glycosylation and mitochondrial antioxidant defense results in a devastating multisystem phenotype [3, 6].

Since its initial description by Park et al. in 2015 [1], fewer than 20 patients with SLC39A8‐CDG have been reported worldwide [7–9]. The clinical spectrum includes severe developmental delay, hypotonia, seizures, dystonia, cranial dysmorphism, dwarfism, hearing loss, and visual impairment [1, 6, 7]. Neuroimaging characteristically shows bilateral T2‐hyperintense basal ganglia lesions mimicking Leigh syndrome, frequently accompanied by cerebral and cerebellar atrophy [6, 7, 10]. Importantly, SLC39A8‐CDG is a treatable condition: oral manganese and galactose supplementation can correct the glycosylation defect and produce clinical improvement [11, 12].

## 2. Case Presentation

An 11‐month‐old female infant was referred to us with new‐onset unprovoked seizures and developmental regression. She was born at term to nonconsanguineous, clinically healthy parents. Pregnancy and the postnatal course were uneventful. Early development was reported as age‐appropriate until approximately 6 months, when the parents noted progressive loss of previously acquired motor and social milestones. Development was reportedly normal during the first 6 months of life, with attainment of age‐appropriate milestones, including social smiling, good head control, reaching for and grasping objects, rolling over, visual tracking, and appropriate social engagement. At approximately 6 months of age, she experienced an acute developmental regression with loss of previously acquired skills. The regression progressed rapidly, and by 11 months of age she had lost all previously attained milestones. At the time of assessment, she no longer demonstrated social smiling, visual fixation or tracking of objects, or head control, consistent with profound global neurodevelopmental regression.

On examination, she demonstrated global hypotonia, poor visual engagement, and weak sucking with consequent poor feeding and faltering growth. The deep tendon reflexes were diminished. No dysmorphic features, hepatosplenomegaly, or skin abnormalities were observed. Family history was noncontributory.

Her EEG was normal, brain magnetic resonance imaging (MRI) revealed bilateral, symmetric T2 hyperintensities in the lentiform nuclei (putamina and globi pallidi) and cerebral peduncles (Figure 1). These lesions showed restricted diffusion on diffusion‐weighted imaging, which was consistent with cytotoxic oedema. T1‐weighted sequences showed isointense signal with no abnormal postcontrast enhancement. Prominent extra‐axial CSF spaces were noted frontally and temporally, consistent with mild cerebral volume loss or benign external hydrocephalus. Gray white matter differentiation was otherwise preserved, and the cerebellum was structurally normal. This pattern of symmetric basal ganglia and brainstem T2 hyperintensities with diffusion restriction is characteristic of SLC39A8‐CDG [6, 7, 10] and closely mimics Leigh syndrome [13, 14].

**FIGURE 1:**
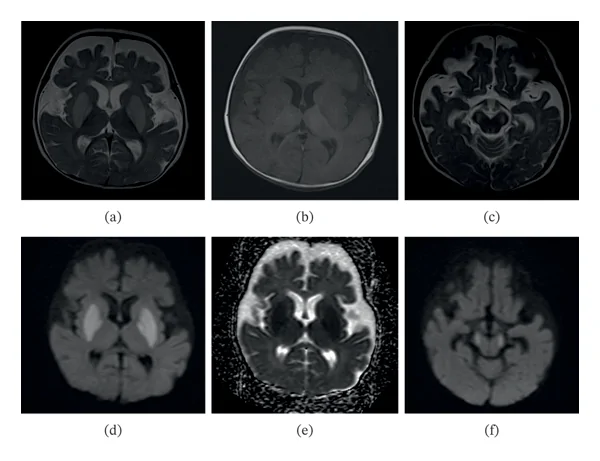
Bilateral symmetric T2 hyperintense signals in the lentiform nuclei (a) isointense on T1 (b) T2 hyperintense signals in bilateral cerebral peduncles (c), and diffusion restriction in bilateral lentiform nuclei and cerebral peduncles (d–f).

The differential diagnosis of bilateral symmetric basal ganglia T2 hyperintensities in infancy includes:

• Leigh syndrome is the principal clinical mimic, caused by mitochondrial respiratory chain defects with elevated CSF lactate [13, 14]. SLC39A8‐CDG has been termed “Leigh‐like” owing to overlapping imaging and secondary mitochondrial dysfunction [6].
• Biotin‐thiamine‐responsive basal ganglia disease (BTBGD) is caused by biallelic SLC19A3 variants and is characterized by acute/subacute encephalopathy with bilateral caudate and putaminal lesions that are treatable with high‐dose biotin and thiamine [15, 16]. This is a critical differential, as it is also a treatable condition.
• Organic acidurias (e.g., glutaric aciduria Type I and methylmalonic acidemia) are typically distinguished by urine organic acid profiles.
• Other manganese transportopathies SLC30A10 and SLC39A14 deficiency cause excess manganese (hypermanganesemia with dystonia), the opposite metabolic derangement [2, 17].

Biochemical and metabolic evaluation: Table 1 shows the detailed metabolic and biochemical evaluation performed, which was not suggestive of any specific etiology.

**TABLE 1:** Biochemical investigations.

Test	Result	Repeat labs	Units
Glucose	95		mg/dL
Magnesium	2.6		mg/dL
Creatinine	0.5		mg/dL
Sodium (initial)	136	140	mmol/L
Potassium (initial)	4.1	4.6	mmol/L
Chloride (initial)	98	104	mmol/L
HCO_3_ (serum; initial)	7.1	17.2	mmol/L
Calcium	9.3		mg/dL
CBC	Unremarkable		Qualitative
Ammonia (NH_3_)	70		μmol/L
Blood gas pH	7.39		—
Blood gas PCO_2_	15		mmHg
Blood gas PO_2_	110		mmHg
Blood gas HCO_3_	9.1		mmol/L
Blood gas base excess	−12.8		mmol/L
Lactate (initial)	10.6		mmol/L
Homocysteine	5.4		μmol/L
Plasma amino acids	Nondiagnostic		Qualitative
Urine organic acids (UOA)	Nondiagnostic		Qualitative
TSH	0.3		μIU/mL
Free T4 (FT4)	1.29		ng/dL
SGPT/ALT	8		U/L
Lactate (repeat)	3.0		mmol/L
CSF lactate (paired with repeat lactate)	2.31		mmol/L
CPK	397		U/L
CSF detailed report (CSF DR)	Unremarkable		Qualitative

We referred her for genetic testing. Whole exome sequencing identified a novel homozygous missense variant in SLC39A8: NM_001,135,146.2:c.1106A > *C* (p.Asn369Thr). The variant resulted in the substitution of a highly conserved asparagine residue with threonine at position 369 of the ZIP8 transporter protein. This variant was absent from ClinVar and gnomAD databases. Both parents were confirmed to be heterozygous carriers, with parental identity verified, consistent with autosomal recessive inheritance [2, 6]. In silico prediction tools (SIFT, PolyPhen‐2, and aCADD) predict a deleterious effect, although the variant is currently classified as a variant of uncertain significance (VUS) per ACMG/AMP criteria [18]. Exome sequencing was performed on an MGI T7 platform, targeting a mean depth of 100–120×; the reported mean depth was 119×, with 99.5% of target bases covered at ≥ 20×. Reads were aligned to GRCh38 using BWA‐MEM v0.7.17, and variants were annotated using Ensembl VEP. A heterozygous SLC39A8 variant, NM_001,135,146.2:c.1106A > *C* (p.Asn369Thr), chr4:102,267,617 T > *G* (GRCh38), was reported. The laboratory listed ACMG criterion PM2 and classified it as a ‘VUS’; in silico predictions were REVEL 0.64 (deleterious), PrimateAI 0.73 (uncertain), AlphaMissense 0.744 (uncertain), SIFT 0.001 (uncertain), and SpliceAI 0 (benign). The gnomAD v4.1.0 allele frequency and ClinVar classification were reported as unavailable.

At the time of reporting, confirmatory metabolic investigations of serum transferrin isoelectric focusing and blood manganese levels were planned but could not be sent because these tests are unavailable. If a Type II transferrin isoform pattern and low blood manganese levels are confirmed, targeted therapy with oral manganese sulfate supplementation and oral d‐galactose should be initiated under specialist metabolic supervision [11, 12]. Empirical biotin and thiamine were considered to exclude BTBGD pending genetic results [15, 16].

The patient remains under close multidisciplinary follow‐up including pediatric neurology, clinical genetics, and metabolic medicine. Antiepileptic medications were initiated. Formal ophthalmological and audiological assessments have been performed, given the known association of SLC39A8‐CDG with cortical visual impairment and sensorineural hearing loss [1, 6]. The parents received genetic counselling regarding the 25% recurrence risk for future pregnancies, and carrier testing was recommended for extended family members.

## 3. Discussion

First, p.Asn369Thr adds to the catalogue of reported SLC39A8 variants, but its pathogenicity remains uncertain in this patient because it is currently a VUS. Previously reported variants included p.Gly38Arg, p.Cys113Ser, p.Ile340Asn, p.Gly204Cys, p.Ser335Thr, p.Ala391Thr, p.Phe203Ser, p.Gly350Arg, and p.Ser44Trp [1, 6–8]. Functional studies of some disease‐associated ZIP8 variants have reported defective localization and Mn uptake [3]. Those findings cannot be assumed for p.Asn369Thr; dedicated functional evidence and/or additional case evidence would be needed for reclassification.

Second, the MRI pattern in this patient (bilateral basal‐ganglia signal abnormality with brainstem extension and dentate sparing) supports a Leigh‐like differential diagnosis but is not specific for SLC39A8‐CDG. Riley et al. [6] described siblings with homozygous SLC39A8 p.Cys113Ser and basal‐ganglia T2 hyperintensities with cerebral atrophy. Bonaventura et al. [7] reported a Leigh‐like MRI pattern in patients with SLC39A8 variants. Conventional transferrin glycosylation analysis may be normal in some affected patients; where clinically available, mass‐spectrometry‐based N‐glycome analysis may be more sensitive [8].

Third, SLC39A8‐CDG has potentially treatable biochemical features, and published reports have described biochemical and clinical improvement after manganese and/or galactose‐containing regimens [6, 11, 12]. These reports do not establish efficacy or safety for the present infant with an unconfirmed diagnosis. Manganese supplementation should therefore be initiated only after specialist metabolic assessment, with biochemical monitoring and attention to potential manganese toxicity.

Fourth, a VUS does not independently establish a molecular diagnosis. In this case, the phenotype, MRI pattern, gene‐disease relationship, and reported parental carrier status provide supportive but not definitive evidence. Metabolic findings, the complete laboratory classification evidence, and functional testing of p.Asn369Thr would help refine the interpretation.

## 4. Conclusion

SLC39A8‐related CDG (SLC39A8‐CDG; CDG type IIn) should be considered in infants presenting with developmental regression, seizures, and bilateral symmetric basal ganglia abnormalities on brain MRI, particularly when the imaging findings resemble Leigh syndrome. T2‐weighted hyperintensities involving the caudate nuclei, putamina, globi pallidi, and brainstem, with relative sparing of the dentate nuclei, may support this consideration. Biochemical investigations, including blood manganese measurement and transferrin glycosylation analysis, are recommended to help establish the diagnosis.

Although the clinical and radiological findings raise suspicion for SLC39A8‐CDG, they are not diagnostic. The reported SLC39A8 variant remains a VUS, and its clinical relevance should be assessed alongside the phenotype, biochemical findings, and any available functional evidence. Further metabolic evaluation and functional studies may help clarify the diagnosis and inform potential variant reclassification.

Given the potential therapeutic benefit of manganese and galactose supplementation in confirmed SLC39A8‐CDG, the possibility of initiating targeted supplementation in cases with a high clinical suspicion but inconclusive diagnostic investigations should be discussed with an experienced metabolic specialist. Any decision to initiate treatment before definitive confirmation should be individualized, taking into account the clinical presentation, biochemical findings, available evidence supporting the diagnosis, and the potential benefits and risks of treatment. Appropriate monitoring of manganese levels, treatment response, and potential toxicity is essential.

## Author Contributions

Conceptualization was carried out by Muhammad Nasheet Sagri, Bushra Rehman, and Abida Akbar, while formal analysis was performed by Abida Akbar and Muhammad Nasheet Sagri. The original draft of the manuscript was written by Abida Akbar, Zohra Hasan, and Muhammad Nasheet Sagri, and the review and editing process involved contributions from Khairunnisa Muhktiar, Salman Kirmani, and Bushra Rehman.

## Funding

This study received no specific grant from any funding agency in the public, commercial, or not‐for‐profit sectors.

## Ethics Statement

Written informed consent was obtained from the parent of the patient for publication of this case report, which did not require formal full review.

## Consent

Please see the Ethics Statement.

## Conflicts of Interest

The authors declare no conflicts of interest.

## Data Availability

The data supporting the findings of this study are available from the corresponding author upon reasonable request.
